# Locus of Control and Leader–Member Exchange: A Dimensional, Contextualized, and Prospective Analysis

**DOI:** 10.3389/fpsyg.2020.537917

**Published:** 2020-10-23

**Authors:** Véronique Robert, Christian Vandenberghe

**Affiliations:** Department of Management, HEC Montréal, Montréal, QC, Canada

**Keywords:** locus of control, leader–member exchange, role clarity, leadership, dimensional approach, moderation analysis

## Abstract

Since the relationship between leaders and subordinates has important implications for organizations, exploring how high-quality leader–member exchange (LMX) relationships develop over time is a critical research objective. However, LMX research has essentially focused on leader-centric approaches to describe how leaders develop differential relationships with subordinates and has devoted little attention to the influence of subordinate characteristics. This study contends that subordinates’ individual differences may act as drivers of LMX relationships. Specifically, we posited that individuals with an internal work locus of control, owing to their sense of control over the work environment, are more prone to develop high LMX relationships over time. Moreover, we expected this effect to be enhanced when these individuals are given clear expectations about their work role because such conditions would ease their sense of agency. Further, we suggested that these effects may partly depend on the dimension of LMX (i.e., affect, loyalty, contribution, and professional respect) under consideration. We argued that the effect of internal work locus of control would generalize to all LMX dimensions but that its interaction with role clarity would primarily impact the loyalty and contribution dimensions of LMX as their behavioral orientation would result in valued outcomes for internals. Data were collected through questionnaires among a sample of 424 employees working in various industries. Through a two-wave study and controlling for the autoregressive effects of LMX, subordinates’ internal work locus of control was found to enhance LMX relationships over time. Using a multidimensional approach to LMX, our results further show that the effect of internal work locus of control generalized to all dimensions of LMX. Using a contextualized view of the development of LMX, we also found that role clarity moderated the positive relationship between internal work locus of control and LMX over time such that the relationship was stronger when role clarity was high. However, from a dimensional perspective, role clarity only accentuated the relationship between work locus of control and LMX’s loyalty dimension. The implications of these findings for LMX research are discussed.

## Introduction

The leader–member exchange (LMX) theory stipulates that supervisors engage in relationships of distinct quality with subordinates depending on how interactions develop within each employee–supervisor dyad (Graen and Scandura, 1987; Liden et al., 1993; Graen and Uhl-Bien, 1995). High-quality LMX is characterized by social exchange relationships that give way to mutual trust, commitment, reciprocity, and loyalty among members of the dyad. In these situations, subordinates receive resources, rewards, and challenging job assignments that help them develop and be efficient in their work role (Liden et al., 1997). As such, high LMX reflects a relational context where socioemotional exchanges are ubiquitous (Dansereau et al., 1975; Vidyarthi et al., 2010). In contrast, low-quality LMX is characterized by economic exchange based on give and take inputs where transactions are limited to the terms of tangible employment contracts (Blau, 1964; Liden et al., 1993). There has been abundant research showing that high-quality LMX is associated with a host of positive outcomes including heightened organizational commitment, job performance, and organizational citizenship behaviors (e.g., Gerstner and Day, 1997; Anand et al., 2011; Dulebohn et al., 2012; Rockstuhl et al., 2012; Martin et al., 2016).

A significant number of studies have also been devoted to identifying the antecedents of LMX. These antecedents have been categorized into follower characteristics, leader characteristics, characteristics of the interpersonal relationship with the leader, and contextual variables (Dulebohn et al., 2012). Among these antecedents, less attention has been devoted to follower characteristics, and when these characteristics were investigated, the studies mostly focused on the dispositional traits of positive and negative affectivity (Dulebohn et al., 2012; Tse et al., 2018). In this research, we focus on employees’ work locus of control as a driver of LMX. As a personality trait, locus of control reflects a relatively stable belief that the environment can either be influenced (i.e., internal locus of control) or that events are driven by chance or fate (i.e., external locus of control) (Rotter, 1966). Because it affects how individuals interpret events and the way they act across multiple situations (Rotter, 1966), locus of control provides important insights into human behavior in organizations (Spector, 1982). Rooted in Rotter’s (1954) social learning theory, the locus of control represents the implicit expectancies that individuals hold regarding their ability to obtain valued outcomes through their own actions (Rotter, 1966; Lefcourt, 1976; Wang et al., 2010). Internals view themselves as masters of their own fate and have strong behavior-reward expectancies (Rotter, 1966; Spector, 1982; Johnson et al., 2015) while externals view their lives as being governed by external forces and have a low sense of agency (Ng et al., 2006; Galvin et al., 2018). As a context-specific trait, work locus of control represents the extent to which individuals believe that the rewards they obtain at work result from their actions (Spector, 1988; Harris et al., 2007; Wang et al., 2010). Individuals who possess an internal work locus of control have better interpersonal skills in the work context and are more socially astute and able to influence others (Ng et al., 2006; Wang et al., 2010). Therefore, one can expect them to develop higher LMX relationships with their supervisor. However, although previous research has empirically examined the relationship between locus of control and LMX, this research has been cross-sectional (e.g., Martin et al., 2005; Harris et al., 2007; Kauppila, 2014; Hao et al., 2019), providing no evidence for a longitudinal effect. As we explain below, demonstrating that work locus of control predicts that LMX is an important endeavor that elucidates its role as a driver of LMX development. The first goal of our study is thus to extend the current line of work and examine whether work locus of control drives change in LMX over time.

Second, this study aims to explore a contextualized view of the contribution of locus of control to LMX. Specifically, we introduce role clarity as a boundary condition in this relationship. Role clarity refers to situations where role expectations are clearly defined and specified to employees (Rizzo et al., 1970). Such situations may be appealing to internals. Indeed, the locus of control literature suggests that internals have a strong need for achievement (Yukl and Latham, 1978; Spector, 1982), feel intrinsically motivated to achieve desired goals (Ng et al., 2006), and are confident in the instrumentality of their efforts to achieve performance goals (Spector, 1982). Following this reasoning, we posit that internals will feel more confident in their ability to influence their environment (e.g., the relationship with supervisors) when the expectations regarding their role are clearly specified (Lam and Schaubroeck, 2000). In other words, when role expectations are clearer, internals may perceive that the link between their actions and the outcomes they obtain (e.g., LMX) is stronger, thereby increasing their sense of agency. Thus, we propose that the contribution of work locus of control to LMX will be stronger when role clarity is high.

Third, the present study purports to look at LMX at both a construct level and a dimension level. Liden and Maslyn (1998) conceived LMX as a multidimensional construct. Their measure encompasses four dimensions: affect, loyalty, contribution, and professional respect. However, although their measure and approach have been largely endorsed among LMX scholars, research has primarily adopted a unitary view of LMX (Dulebohn et al., 2017). Therefore, the examination of the antecedents, correlates, and outcomes of LMX at the construct level tends to ignore that associations may vary across LMX dimensions. This may be problematic as such practice assumes that the relationships between LMX and other constructs in its nomological network are homologous at the construct and dimension levels (Wong et al., 2008). To account for this potential discrepancy, this study will examine whether the proposed main and interactive effects of work locus of control on change in LMX across time generalize from the construct level to the dimension level. As work locus of control is thought to lead to stronger behavior-reward expectancies, we reason that an internal work locus of control should particularly impact LMX’s behavioral dimensions (i.e., loyalty and contribution) when role clarity is high.

The present study contributes to the LMX literature from three perspectives. First, by examining work locus of control as a driver of LMX over time, we break new ground by suggesting that individuals’ interpersonal skills and dispositional capacity to influence others make some employees more likely to develop high-LMX relationships with supervisors. As such, this study counterbalances the dominant perspective that LMX relationships are bound to parties’ willingness to engage in social exchange relationships (e.g., Anand et al., 2010, 2018; Lord et al., 2016). This counterbalanced view suggests that employees’ dispositions may play a role in how LMX develops from its early stages (Graen and Scandura, 1987; van Breukelen et al., 2006). Moreover, to demonstrate that these effects hold over time, we used a multiwave design that controlled the autoregressive effects of LMX and its dimensions, which answers the call of researchers to use designs that allow examining longitudinal relationships between antecedent variables and LMX (e.g., Dulebohn et al., 2012; Martin et al., 2016). Second, this study promotes a contextualized view of the relationship between work locus of control and LMX. As locus of control “functions in part as an evaluation of the environment” (Johnson et al., 2015, p. 1570), its effects are likely stronger in environments that provide opportunities to reinforce people’s agency (Galvin et al., 2018). Therefore, this study is a plea for conceiving LMX development as the result of the joint influence of the person and situation (Ozer, 2008). Finally, by studying the proposed relationships at LMX’s construct and dimension levels, this study underlines the importance of considering the dimensions of LMX as separate components of a social exchange relationship (Maslyn and Uhl-Bien, 2001; Sin et al., 2009) that may differ in responsiveness to work locus of control and role clarity.

## Theoretical Background and Hypotheses

### Work Locus of Control and LMX: A Construct Level Perspective

As a relationship-based model of leadership (Graen and Uhl-Bien, 1995; Liden et al., 1997), LMX reflects the quality of social exchange relationships between employees and supervisors. One of the fundamental assumptions of the model is that leaders develop relationships of a distinct quality with different subordinates (Wayne et al., 1997; Schriesheim et al., 1999; Martin et al., 2005). High LMX reflects situations where the exchange relationship is based on mutual trust and commitment, with subordinates benefiting from intrinsic rewards, challenging assignments, and opportunities to grow, while low LMX refers to situations where economic exchange relationships represent the rule, and subordinates complete their duties in exchange for a given salary and tangible assets (Dienesch and Liden, 1986; Graen and Scandura, 1987; Wayne et al., 1997).

Among individual traits that have been studied as antecedents to LMX, positive and negative affectivity and the Big-Five personality trait of extraversion have attracted the most attention (Dulebohn et al., 2012). While less studied as a predictor of LMX, the locus of control has nonetheless characteristics that make it a relevant antecedent. In this study, we focus on work locus of control because it is a domain-specific personality measure that reflects the individual’s chronic perception of control within the work context and as such may exhibit improved validity over general locus of control (Lievens et al., 2008; Wang et al., 2010). People with an internal work locus of control tend to attribute the rewards they obtain at work to their personal efforts (Spector, 1988) and generally perceive their efforts as being instrumental to obtaining rewards and attaining valued goals (Lam and Schaubroeck, 2000). They also have a strong need for achievement, indicating that they strive to meet the standards of performance prevailing in their workplace, seek personal growth and learning opportunities, and have a sense of agency in obtaining positive outcomes from their environment (Spector, 1982; Allen et al., 2005; Ozer, 2008). Supporting this view, meta-analytic results indicate that internals obtain higher salaries and experience heightened career satisfaction (Ng et al., 2005).

As supervisors are agents that represent the organization (Erdogan and Enders, 2007) and influence the promotion and reward decisions that apply to subordinates (Webster and Beehr, 2013), the quality of exchange relationships with them is critical for those who want to get ahead in the organization. For instance, research has found LMX relationships to be positively associated with promotability ratings (Scandura and Schriesheim, 1994; Wayne et al., 1999), particularly when relationship or organizational tenure is high (Harris et al., 2006). Research also indicates that strong LMX relationships help subordinates gain influence in the organization’s network (Sparrowe and Liden, 2005). These results suggest that developing high LMX relationships is a useful means for getting ahead and pursuing career goals in the organization. As locus of control typically involves a social learning process whereby the individual identifies the events and behaviors that are causally related to valued rewards (Rotter, 1966; Galvin et al., 2018), internals may perceive more quickly than externals that a good LMX relationship is a milestone toward desired outcomes and that their behavior can make a difference in developing LMX. Moreover, as internals cultivate interpersonal relationships (Ng et al., 2006) and are more effective than externals to influence others (Wang et al., 2010), they are likely to generate more positive relationships with their supervisors. Thus, we expect an internal work locus of control to drive LMX. This effect should also persist over time (i.e., longitudinally) because internals maintain consistent expectancies that their relational environment is responsive to their actions, resulting in cumulative reinforcements (Galvin et al., 2018). The above discussion leads to the following hypothesis.

*Hypothesis 1*: Internal work locus of control will be positively related to change in LMX over time.

### Work Locus of Control and LMX: A Dimension Level Perspective

Numerous studies have adopted a unidimensional view of LMX (Dulebohn et al., 2017). However, it is likely that the nature of overall LMX reflects different combinations of its dimensions depending on circumstances (Liden and Maslyn, 1998; Maslyn and Uhl-Bien, 2001). Unfortunately, the few studies that have explored LMX dimensions separately have focused on their consequences (for exceptions, see Maslyn and Uhl-Bien, 2001; Lee, 2005) instead of their antecedents (e.g., Greguras and Ford, 2006). According to role theory, roles are inherently multidimensional (Katz and Kahn, 1978). Thus, people may endorse different roles in the workplace (Liden and Maslyn, 1998) and depending on what roles are salient in exchange relationships, different types of LMX are likely to emerge (Dienesch and Liden, 1986; Liden et al., 1997; Liden and Maslyn, 1998). The multidimensional approach to LMX (Liden and Maslyn, 1998) acknowledges that members may take on different roles, leading to different currencies of exchange being salient to LMX (Dienesch and Liden, 1986; Law et al., 2010) depending on the resources and opportunities that are valued by the dyad members (Graen and Cashman, 1975).

Liden and Maslyn’s (1998) multidimensional framework identifies four dimensions within LMX: affect, loyalty, contribution, and professional respect. *Affect* refers to the mutual affection that LMX partners feel for one another. Such affection is driven by interpersonal attraction and is essentially an attitude toward the other member of the dyad. Internals are known to develop friendly relationships with others and to cultivate constructive social relationships (Ng et al., 2006). Building an affect-based relationship with the supervisor may help internals get access to resources from the supervisor and attain their desired goals. For example, internal work locus of control has been found to be positively related to leader consideration and social support at work, and to be negatively related to interpersonal conflict at work (Wang et al., 2010). By extension, one may expect internal work locus of control to be positively associated to LMX’s affect dimension (i.e., LMX-Affect). Again, one may expect this relationship to hold over time as internals maintain a consistent perception over time that the environment (e.g., the supervisor) is responsive to their actions (Galvin et al., 2018). This leads to the following hypothesis.

*Hypothesis 2a*: Internal work locus of control will be positively related to change in LMX-Affect over time.

*Loyalty* (e.g., LMX-Loyalty) is the second dimension of LMX. It refers to the expression by one member (e.g., the supervisor) of public support for the other member, his or her goals, and character (Liden and Maslyn, 1998). An example of item measuring this dimension is “My supervisor would defend me to others in the organization if I made an honest mistake” (Liden and Maslyn, 1998, p. 56). Loyalty differs from the socioemotional dimension of affect as it is behavioral in nature and refers to the dyadic members’ concrete behaviors that manifest support to each other. Through social learning, internals acquire implicit knowledge regarding what actions may help them earn the support of powerful others such as supervisors (Rotter, 1966; Spector, 1982, 1988). As loyalty reflects behavioral support, it represents a strong manifestation of the outcomes pursued by internals. Moreover, internals may themselves be loyal to their supervisors because this can help them achieve desired goals. For example, leaders were found to be more likely to ask loyal members to take on tasks that required independent judgment and responsibility (Liden and Graen, 1980). Therefore, being loyal to the leader allows individuals with an internal locus of control to benefit from more autonomy in carrying out their tasks (Liden and Maslyn, 1998), which is a central concern for internals (Ng et al., 2006). Thus, we propose the following hypothesis from a longitudinal perspective.

*Hypothesis 2b*: Internal work locus of control will be positively related to change in LMX-Loyalty over time.

The third dimension of LMX, *contribution* (i.e., LMX-Contribution), refers to the perception by members of the dyad that each member engages in work activities that benefit the mutual goals of the dyad (Liden and Maslyn, 1998). As loyalty, contribution is behavioral in nature: it reflects actions undertaken by the members of the dyad that help the attainment of the dyadic goals. LMX-Contribution also involves the completion by subordinates of tasks that go beyond their job description and the facilitation of such activities by the supervisor. Since internal work locus of control is a task-related construct (Henson and Beehr, 2018) that is positively related to job performance (Ng et al., 2006; Wang et al., 2010), individuals with internal work locus of control are bound to invest energies in activities that exceed expectations, thereby fostering LMX-Contribution (Maslyn and Uhl-Bien, 2001). They likely contribute time and energies to develop the relationship with their supervisor because internals have a strong need for achievement, put a premium on performance, and set difficult goals (Yukl and Latham, 1978; Spector, 1982). Moreover, this relationship should be sustained over time. Thus, the following hypothesis is proposed.

*Hypothesis 2c*: Internal work locus of control will be positively related to change in LMX-Contribution over time.

*Professional respect* is the fourth and last dimension of LMX. Contrary to the other dimensions, it has a more contemplative foundation as it connotes expert power and that “each member of the dyad has built a reputation” (Liden and Maslyn, 1998, p. 50) and excels in his or her work. Supervisors possessing such qualities may be perceived by subordinates as powerful and being able to facilitate career success in the organization by connecting subordinates to the larger organization’s network. As such, individuals with an internal work locus of control may be tempted to develop effective relationships with such powerful supervisors because it provides more opportunities to access the desirable resources they pursue. They may also gain knowledge and skills as a result of close interactions with a respected supervisor (Liden and Maslyn, 1998), which would help them achieve better performance. Thus, we propose the following hypothesis, which is framed within a longitudinal perspective.

*Hypothesis 2d*: Internal work locus of control will be positively related to change in LMX-Professional respect over time.

### The Moderating Role of Role Clarity

The extent to which employees are given clearly defined jobs and receive sufficient information to effectively fulfill their role (Rizzo et al., 1970) is an important means by which the work context exerts influence on employees. The variations in employees’ work role expectations are captured by role clarity, which refers to the sufficiency of information provided to employees regarding the expectations associated with their role in the organization (Kahn et al., 1964). Thus, it represents the extent to which such expectations are fully understood by employees (Rizzo et al., 1970). Role clarity is often attributed to the supervisor because he or she assigns the goals and the responsibilities associated with the employee’s role (Panaccio and Vandenberghe, 2011). When role clarity is high, employees understand what is expected of them and are cognizant of the available means to carry out their job tasks, while in situations of low role clarity, employees lack an understanding of what is expected of them, hence, of what it takes to attain performance goals (Newman et al., 2015).

All LMX relationships are contextualized, meaning that situational factors may influence the development of exchange relationships with the supervisor (Liden et al., 1997; Nahrgang and Seo, 2015). For instance, it may be more difficult for employees to exert energies in developing favorable relationships with their supervisor when they have to continuously struggle to understand what is expected of them and to find the appropriate manner in which tasks must be completed. Such situations may be particularly frustrating as employees tend to attribute the responsibility of them to supervisors. Moreover, low role clarity would limit the ability of employees to match appropriate behaviors to role requirements, resulting in lower performance (Tubre and Collins, 2000). In such situations, employees would focus on trying to understand the key responsibilities of their jobs instead of investing energies in developing the relationship with the supervisor. The reverse would be true when role clarity is high. When role expectations are clear and understood, employees may feel confident in finding their ways to get their job done. On empirical grounds, role clarity has been found to be positively related to LMX (Gerstner and Day, 1997; Dulebohn et al., 2012; Gregersen et al., 2016).

We posit that role clarity will also moderate the relationship between work locus of control and LMX. Indeed, it is likely that internals will feel more in control of their environment when role clarity is high (Wang et al., 2016). As internals have a strong need for achievement, their sense of agency and influence will be heightened when job expectations are clearly defined. As Spector (1982) noted, internals actively seek and function better in environments where control is achievable. Therefore, we suggest that the perception of causality between internals’ actions and outcomes such as LMX will be increased when role expectations are clearly communicated, leading to a stronger relationship between internal work locus of control and LMX when role clarity is high. This leads to the following hypothesis.

*Hypothesis 3a*: Internal work locus of control will interact with role clarity such that it will be more (vs. less) strongly related to LMX over time when role clarity is high (vs. low).

Looking at LMX at the dimension level, it seems likely that role clarity moderates the relationship between internal work locus of control and specific LMX dimensions. We specifically posit that the association between internal work locus of control and the *behavioral* dimensions of LMX (i.e., loyalty and contribution) is more likely to be subject to moderating effects by role clarity. Indeed, one may expect that when job responsibilities are clearly specified (i.e., high role clarity), internals will be particularly able to obtain *behavioral inputs* from their supervisor [i.e., having supervisors publicly defending the employee (loyalty)] and engage themselves in *behavioral dedication* to the relationship (contribution). LMX-Loyalty and LMX-Contribution are indeed tangible outcomes, while the other LMX dimensions reflect an attitude (affect) and a contemplative judgment (professional respect) that represent objective outcomes less sought after by internals. In sum, a context of clear job responsibilities allows internals to obtain tangible indications of support by their supervisor (loyalty) and demonstrate dedication to contribute above and beyond job requirements to the relationship with the supervisor (Liden and Maslyn, 1998). This is because a clear definition of their role helps internals obtain and demonstrate indications of desired outcomes, which we expect to hold over time. Thus, we propose the following, remaining hypotheses.

*Hypothesis 3b*: Internal work locus of control will interact with role clarity such that it will be more (vs. less) strongly related to change in LMX-Loyalty over time when role clarity is high (vs. low).
*Hypothesis 3c*: Internal work locus of control will interact with role clarity such that it will be more (vs. less) strongly related to change in LMX-Contribution over time when role clarity is high (vs. low).

## Materials and Methods

### Procedure

Participants were recruited through the personal contacts of the research team, the university’s research panel, and the alumni association’s mailing list, in Eastern Canada. They were asked to participate in a three-wave study about leadership and workplace attitudes. Data for this study were from Time 1 and Time 2. To participate in the study, respondents were to be employed, aged 18 or more, and were to have an identifiable supervisor. Participants were informed that their participation was voluntary and were assured of the confidentiality of their responses. Responses were collected through online surveys using a 6-month time lag. To encourage participation, respondents were given a $5 gift card upon completion of each wave of the surveys. They completed a French or an English version of the questionnaires. Work locus of control, role clarity, LMX, and demographics were measured at Time 1, while LMX was also measured at Time 2. To strengthen the robustness of our design, our analyses controlled for the autoregressive effect of the dependent variable at Time 1 (i.e., overall LMX or LMX dimensions) when examining the effects of the independent variable (e.g., work locus of control) and the moderator (e.g., role clarity) on Time 2 overall LMX or LMX dimensions.

### Sample

Initially, 1,003 participants completed the Time 1 questionnaire, among whom careless respondents (*n* = 3) were excluded, and 655 participants completed the Time 2 questionnaire (for a 66% response rate). To determine if participant attrition was randomly distributed, we conducted a logistic regression analysis to evaluate if Time 1 variables (i.e., work locus of control, role clarity, LMX, LMX-Affect, LMX-Loyalty, LMX-Consideration, and LMX-Professional respect) and demographics (i.e., gender, age, organizational tenure, and tenure with supervisor) influenced the probability of participating at Time 2 (Goodman and Blum, 1996). The logistic regression model was significant [χ^2^(11) = 31.77, *p* < 0.01]. However, none of the predictors were significant in the equation, suggesting that attrition over time was randomly distributed.

As our study includes participants who completed both measurement times (*n* = 655) and excludes participants who changed supervisors between Time 1 and Time 2 (*n* = 231), 424 usable responses remained at Time 2. In this final sample, average age was 29.08 years (*SD* = 9.97), and organizational tenure was 4.37 years (*SD* = 5.48). Participants (75% women) have been working with their current supervisor for an average time of 2.36 years (*SD* = 2.85). They worked in various industries, including health care and social assistance (12%), retail trade (12%), finance and insurance (9%), and educational services (8%). They were affiliated with small organizations (<100 employees; 48%), midsize organizations (101 to less than 1,000 employees; 26%), or large organizations (>1,000 employees; 26%). Most participants worked full time (61%) and had at least a bachelor’s degree (72%).

### Measures

All scale items were measured using a five-point Likert-type scale, ranging from 1 (*strongly disagree*) to 5 (*strongly agree*). To create French versions of English scales, a standard translation–back-translation procedure was followed (Schaffer and Riordan, 2003).

#### Work Locus of Control

Participants rated their level of work locus of control at Time 1 using the 16-item scale developed by Spector (1988). This scale includes eight items measuring external work locus of control (e.g., “Getting the job you want is mostly a matter of luck”) and eight items capturing internal work locus of control (e.g., “People who perform their jobs well generally get rewarded for it”). We reversed scores on the external work locus of control items so that a higher score on the overall scale reflected internal work locus of control (for a similar procedure, see Ng et al., 2006; Wang et al., 2010). The internal consistency for this scale was 0.84.

#### Role Clarity

Role clarity was assessed at Time 1 using an adapted version (Panaccio and Vandenberghe, 2011) of Rizzo et al.’s (1970) five-item scale (α = 0.90). A sample item is “I know exactly what is expected of me.”

#### LMX

The 12-item multidimensional scale (LMX-MDM) developed by Liden and Maslyn (1998) was used to measure LMX at Time 1 (α = 0.93) and Time 2 (α = 0.93). In this measure, the four dimensions of LMX are each represented by a three-item scale: affect (e.g., “I like my supervisor very much as a person;” αs = 0.89 at Time 1 and 0.89 at Time 2), loyalty (e.g., “My supervisor would come to my defense if I were “attacked” by others;” αs = 0.86 at Time 1 and 0.90 at Time 2), contribution (e.g., “I do not mind working my hardest for my supervisor;” αs = 0.84 at Time 1 and 0.81 at Time 2), and professional respect (e.g., “I am impressed with my supervisor’s knowledge of his/her job.;” αs = 0.93 at Time 1 and 0.91 at Time 2).

## Results

### Confirmatory Factor Analyses

Confirmatory factor analysis (CFA) through Mplus 7.31 (Muthén and Muthén, 2010) with maximum likelihood (ML) estimation was used to examine the dimensionality of our constructs. Results for CFA models are reported in Table 1. First, as several of our hypotheses considered LMX dimensions separately, we examined the dimensionality of the LMX measure at both Time 1 and Time 2. A four-factor LMX model distinguishing among affect, loyalty, contribution, and professional respect at Time 1 yielded a good fit to the data, χ^2^(48) = 147.43, CFI = 0.97, NNFI = 0.96, RMSEA = 0.070, SRMR = 0.040, and outperformed a one-factor model, χ^2^(6) = 895.72, *p* < 0.001. Similarly, the four-factor LMX model at Time 2 obtained a good fit as well, χ^2^(48) = 171.76, CFI = 0.97, NNFI = 0.96, RMSEA = 0.078, SRMR = 0.041, and proved significantly superior to the one-factor model, χ^2^(6) = 767.22, *p* < 0.001. These results suggest that LMX dimensions can be considered separately.

**TABLE 1:** Fit indices for confirmatory factor analysis models.

Model	χ^2^	*df*	Δχ^2^	Δ*df*	NNFI	CFI	RMSEA	SRMR
**Time 1 LMX**								
(1) Four-factor model	147.43*	48	–	–	0.96	0.97	0.070	0.040
(2) One-factor model	1,043.14*	54	895.72*	6	0.68	0.74	0.208	0.088
**Time 2 LMX**								
(1) Four-factor model	171.76*	48	–	–	0.96	0.97	0.078	0.041
(2) One-factor model	938.98*	54	767.22*	6	0.71	0.76	0.197	0.084
Overall confirmatory factor analysis model								
(1) Theorized 10-factor model	1,166.49*	438	–	–	0.92	0.93	0.063	0.077
(2) Combining Time 1 LMX dimensions	2,153.68*	462	987.19*	24	0.82	0.84	0.093	0.087
(3) Combining Time 2 LMX dimensions	2,037.37*	462	870.88*	24	0.83	0.85	0.090	0.086
(4) Combining Time 1 and Time 2 LMX dimensions	2,742.47*	477	1,575.98*	39	0.76	0.79	0.106	0.091
(5) One-factor model	5,834.26*	483	4667.77*	45	0.45	0.49	0.162	0.121

**N* = 424. NNFI, non-normed fit index; CFI, comparative fit index; RMSEA, root-mean-square error of approximation; SRMR, standardized root mean square residual; LMX, leader–member exchange; ^∗^*p* < 0.01.*

Second, we tested the distinctiveness of our variables (i.e., work locus of control, role clarity, LMX-Affect, LMX-Loyalty, LMX-Contribution, and LMX-Professional respect, at both Time 1 and Time 2) using an overall CFA. The errors of LMX’s parallel items were allowed to correlate across time (Geiser, 2012). In order to simplify the model (Little et al., 2013), the 16 items pertaining to work locus of control were aggregated using a parceling approach. Specifically, items related to external work locus of control were randomly assigned to two parcels and internal work locus of control items were randomly assigned to another two parcels. Results of CFA analyses are presented in Table 1. Our hypothesized 10-factor model yielded a good fit, χ^2^(438) = 1166.49, CFI = 0.93, NNFI = 0.92, RMSEA = 0.063, SRMR = 0.077. Using a nested sequence approach (Bentler and Bonett, 1980), we then compared this model to more parsimonious models. Our hypothesized model outperformed a seven-factor model that either combined Time 1 LMX dimensions [χ^2^(24) = 987.19, *p* < 0.001] or Time 2 LMX dimensions [χ^2^(24) = 870.88, *p* < 0.001], a three-factor model combining Time 1 and Time 2 LMX dimensions [χ^2^(39) = 1,575.98, *p* < 0.001], and a one-factor model [χ^2^(45) = 4,667.77, *p* < 0.001]. Overall, these results suggest that LMX dimensions and all the constructs altogether were distinguishable.

### Descriptive Statistics and Correlations

Descriptive statistics, correlations, and reliability coefficients are presented in Table 2. These correlations are in the expected direction. Time 1 work locus of control was positively related to Time 2 LMX (*r* = 0.33, *p* < 0.01), Time 2 LMX-Affect (*r* = 0.29, *p* < 0.01), Time 2 LMX-Loyalty (*r* = 0.29, *p* < 0.01), Time 2 LMX-Contribution (*r* = 0.26, *p* < 0.01), and Time 2 LMX-Professional Respect (*r* = 0.25, *p* < 0.01). Interestingly, role clarity was also positively related to Time 2 LMX (*r* = 0.25, *p* < 0.01), Time 2 LMX-Affect (*r* = 0.22, *p* < 0.01), Time 2 LMX-Loyalty (*r* = 0.21, *p* < 0.01), Time 2 LMX-Contribution (*r* = 0.14, *p* < 0.01), and Time 2 LMX-Professional Respect (*r* = 0.25, *p* < 0.01).

**TABLE 2:** Means, standard deviations, and correlations among studied variables.

Variable	*M*	*SD*	1	2	3	4	5	6	7	8	9	10	11	12	13	14	15	16
(1) Age (years)	29.08	9.97	–															
(2) Gender (1 = Male; 2 = Female)	1.75	0.43	-0.06	–														
(3) Organizational tenure (years)	4.37	5.48	0.66**	–0.04	–													
(4) Tenure with supervisor (years)	2.36	2.85	0.40**	–0.02	0.57**	–												
(5) LMX (T1)	3.84	0.78	–0.03	0.15**	–0.10	0.05	(0.93)											
(6) LMX-Affect (T1)	3.82	0.95	–0.03	0.13**	–0.06	0.03	0.86**	(0.89)										
(7) LMX-Loyalty (T1)	3.83	0.95	–0.05	0.06	–0.05	0.09	0.85**	0.69**	(0.86)									
(8) LMX-Contribution (T1)	3.92	0.86	–0.03	0.12*	–0.06	0.07	0.75**	0.52**	0.50**	(0.84)								
(9) LMX-Professional respect (T1)	3.79	1.03	–0.05	0.17**	–0.13**	–0.01	0.85**	0.65**	0.62**	0.50**	(0.93)							
(10) Locus of control (T1)	3.61	0.56	0.14**	0.02	0.08	0.07	0.28**	0.27**	0.26**	0.21**	0.18**	(0.84)						
(11) Role clarity (T1)	3.73	0.83	–0.05	0.13**	–0.03	–0.01	0.39**	0.36**	0.33**	0.26**	0.33**	0.19**	(0.90)					
(12) LMX (T2)	3.66	0.83	0.01	0.17**	–0.01	0.07	0.63**	0.61**	0.53**	0.42**	0.53**	0.33**	0.25**	(0.93)				
(13) LMX-Affect (T2)	3.65	1.01	–0.04	0.15**	–0.06	–0.00	0.58**	0.64**	0.48**	0.33**	0.47**	0.29**	0.22**	0.87**	(0.89)			
(14) LMX-Loyalty (T2)	3.66	1.03	–0.01	0.12*	0.03	0.08	0.52**	0.51**	0.55**	0.32**	0.35**	0.29**	0.21**	0.86**	0.70**	(0.90)		
(15) LMX-Contribution (T2)	3.77	0.88	0.08	0.12*	0.05	0.12*	0.42**	0.33**	0.30**	0.48**	0.29**	0.26**	0.14**	0.74**	0.48**	0.53**	(0.81)	
(16) LMX-Professional respect (T2)	3.59	1.07	0.01	0.19**	–0.05	0.04	0.57**	0.53**	0.42**	0.30**	0.61**	0.25**	0.25**	0.86**	0.69**	0.62**	0.51**	(0.91)

*N*s = 422–424. T1, Time 1; T2, Time 2. Reliability coefficients are reported in parentheses along the diagonal. *p < 0.05; **p < 0.01.

### Hypothesis Testing

To test our hypotheses, we conducted a series of multiple regression analyses using SPSS (version 26). We first centered all variables including controls (i.e., Time 1 LMX or LMX dimensions, work locus of control, and role clarity) following Dawson’s (2014) recommendations. Hypothesis 1 predicted that work locus of control would be positively related to Time 2 LMX, controlling for Time 1 LMX. As can be seen in Table 3 (Model 2), controlling for the autoregressive effect of LMX (β = 0.60, *p* < 0.001), Time 1 work locus of control was positively related to Time 2 LMX (β = 0.17, *p* < 0.001). Hypothesis 1 is thus supported. Table 4 reports multiple regression results for LMX dimensions used as separate dependent variables. As can be seen (Table 4, Model 2s), controlling for their respective autoregressive effect (β = 0.61, 0.52, 0.45, and 0.59, all *p* < 0.001, respectively), Time 1 work locus of control was positively related to Time 2 LMX-Affect (β = 0.13, *p* < 0.001), Time 2 LMX-Loyalty (β = 0.16, *p* < 0.001), Time 2 LMX-Contribution (β = 0.17, *p* < 0.001), and Time 2 LMX-Professional respect (β = 0.14, *p* < 0.001). These results provide support for Hypotheses 2a–d.

**TABLE 3:** Results of moderated linear regression analysis for Time 2 overall LMX.

Step	Variable(s) entered	Model 1	Model 2	Model 3	Model 4
(1)	Time 1 LMX	0.64***	0.60***	0.60***	0.60***
	*R*^2^	0.41***			
(2)	Time 1 Locus of control		0.17***	0.17***	0.17***
	Δ*R*^2^		0.03***		
(3)	Time 1 Role clarity			–0.01	–0.00
	Δ*R*^2^			0.00	
(4)	Time 1 Locus of control × Time 1 Role clarity				0.10**
	Δ*R*^2^				0.01**

*LMX, leader–member exchange. Except for Δ*R*^2^ rows, entries are standardized regression coefficients. Final model statistics: Model 1: *F*(1, 420) = 292.67, *p* < 0.001, *R*^2^ = 0.41; Model 2: *F*(2, 419) = 161.86, *p* < 0.001, *R*^2^ = 0.44; Model 3: *F*(3, 418) = 107.69, *p* < 0.001, *R*^2^ = 0.44; Model 4: *F*(4, 417) = 83.71, *p* < 0.001, *R*^2^ = 0.45, ^∗∗^*p* < 0.01; ^∗∗∗^*p* < 0.001.*

**TABLE 4:** Results of the moderated linear regression analyses for Time 2 LMX dimensions.

		T2 LMX-Affect				T2 LMX-Loyalty				T2 LMX-Contribution				T2 LMX-Professional respect			
Step	Variable(s) entered	M1	M2	M3	M4	M1	M2	M3	M4	M1	M2	M3	M4	M1	M2	M3	M4
(1)	T1 LMX-Affect	0.65***	0.61***	0.62***	0.62***												
	T1 LMX-Loyalty					0.56***	0.52***	0.51***	0.52***								
	T1 LMX-Contribution									0.49***	0.45***	0.46***	0.46***				
	T1 LMX-Professional respect													0.62***	0.59***	0.58***	0.58***
	Δ*R*^2^	0.42***				0.31***				0.24***				0.38***			
(2)	T1 Locus of control		0.13***	0.13***	0.13***		0.16***	0.16***	0.16***		0.17***	0.17***	0.17***		0.14***	0.14***	0.14***
	Δ*R*^2^		0.02***				0.02***				0.03***				0.02***		
(3)	T1 Role clarity			-0.02	-0.02			0.01	0.02			-0.01	-0.01			0.04	0.04
	Δ*R*^2^			0.00				0.00				0.00				0.00	
(4)	T1 Locus of control × T1 Role clarity				0.06				0.14***				0.06				0.06
	Δ*R*^2^				0.00				0.02***				0.00				0.00

LMX, leader–member exchange; M1, Model 1; M2, Model 2; M3, Model 3; M4, Model 4. Except for Δ*R*^2^ rows, entries are standardized regression coefficients. Final model statistics, Model 4s: *F*(4, 417) = 80.44, *p* < 0.001, *R*^2^ = 0.44 (T2 LMX-Affect); *F*(4, 417) = 57.29, *p* < 0.001, *R*^2^ = 0.36 (T2 LMX-Loyalty); *F*(4, 417) = 38.25, *p* < 0.001, *R*^2^ = 0.27 (T2 LMX-Contribution); *F*(4, 417) = 70.58, *p* < 0.001, *R*^2^ = 0.40 (T2 LMX-Professional respect), ***p < 0.001.

**FIGURE 1:**
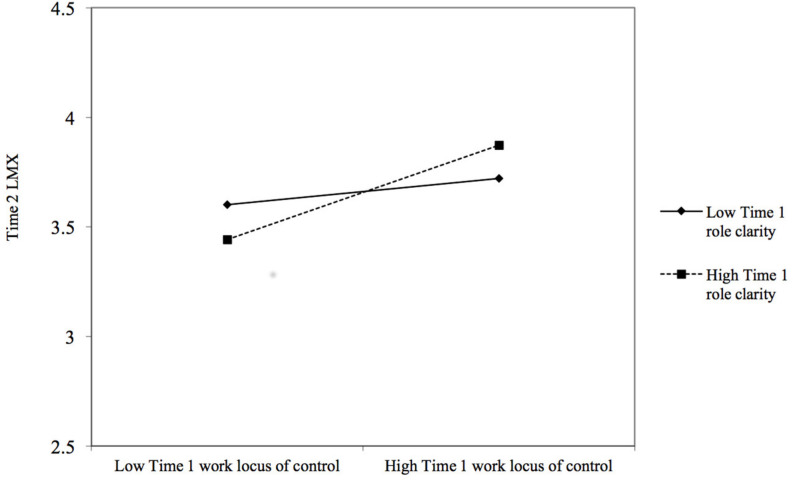
Interaction between Time 1 work locus of control and Time 1 role clarity predicting Time 2 leader–member exchange (LMX), controlling for Time 1 LMX. Relationships are shown at 1 *SD* above and below the mean of Time 1 role clarity.

Hypotheses 3a–c predicted moderating effects of role clarity in the relationship between work locus of control and overall LMX and specific LMX dimensions. Results of the moderated regression analysis for overall LMX are presented in Table 3. We first added centered Time 1 role clarity (Model 3), which was non-significant (β = −0.01, *ns*). However, the interaction between Time 1 work locus of control and Time 1 role clarity (Model 4) significantly predicted Time 2 LMX (β = 0.10, *p* < 0.01; Δ*R*^2^ = 0.01, *p* < 0.01), controlling for Time 1 LMX (β = 0.60, *p* < 0.001). Figure 1 graphically represents the pattern of this interaction, following Aiken and West’s (1991) guidelines. The relationship between Time 1 work locus of control and Time 2 LMX was significantly positive at high levels (1 *SD* above the mean) of Time 1 role clarity [*t*(421) = 5.02, *p* < 0.001], but this relationship was non-significant at low levels (1 *SD* below the mean) of it [*t*(421) = 1.41, *ns*]. The analysis of the regions of significance for this interaction (Preacher et al., 2006) indicated that the relationship between Time 1 work locus of control and Time 2 LMX was significantly positive when Time 1 role clarity exceeded the standardized value of -0.66. Hypothesis 3a is thus supported.

**FIGURE 2:**
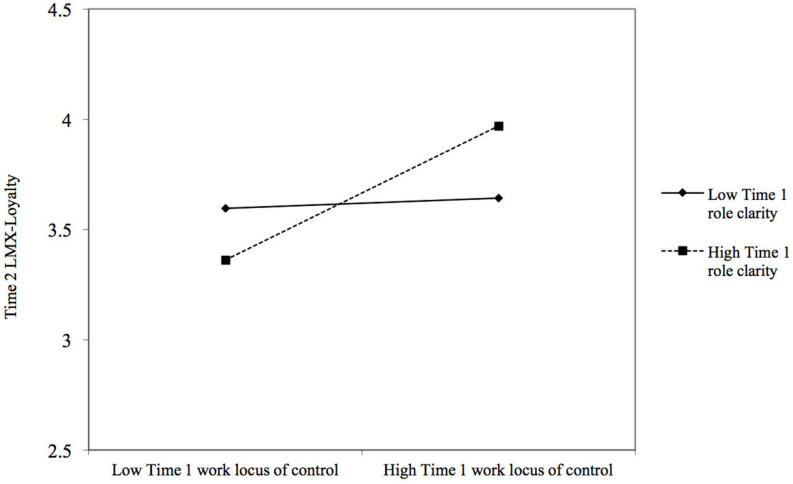
Interaction between Time 1 work locus of control and Time 1 role clarity predicting Time 2 LMX-Loyalty, controlling for Time 1 LMX-Loyalty. Relationships are shown at 1 *SD* above and below the mean of Time 1 role clarity.

Hypotheses 3a–b predicted that Time 1 role clarity would moderate the relationship between Time 1 work locus of control and Time 2 LMX-Loyalty and LMX-Contribution, respectively. Results are reported in Table 4. We first added centered Time 1 role clarity (Model 3s), which was non-significant for both Time 2 LMX-Loyalty (β = 0.01, *ns*) and Time 2 LMX-Contribution (β = −0.01, *ns*). However, as can be seen in Model 4, controlling for Time 1 LMX-Loyalty (β = 0.52, *p* < 0.001), Time 1 work locus of control interacted with Time 1 role clarity to predict Time 2 LMX-Loyalty (β = 0.14, *p* < 0.001; Δ*R*^2^ = 0.02, *p* < 0.001). Figure 2 illustrates the pattern of this interaction. Simple slope tests (Aiken and West, 1991) indicated that the relationship between Time 1 work locus of control and Time 2 LMX-Loyalty was significantly positive [*t*(421) = 5.16, *p* < 0.001] when Time 1 role clarity was high (1 *SD* above the mean), while this relationship was non-significant [*t*(421) = 0.40, *ns*] when it was low (1 *SD* below the mean). The analysis of regions of significance (Preacher et al., 2006) indicated that the relationship between Time 1 work locus of control and Time 2 LMX-Loyalty was significant and positive when Time 1 role clarity exceeded the standardized value of −0.42. Hypothesis 3b is thus supported. In contrast, as shown in Table 4 (Model 4), controlling for Time 1 LMX-Contribution (β = 0.46, *p* < 0.001), Time 1 work locus of control did not interact with Time 1 role clarity to predict Time 2 LMX-Contribution (β = 0.06, *ns*), which disconfirms Hypothesis 3c. Table 4 (Model 4s) also indicates that Time 1 role clarity does not moderate the relationship between Time 1 work locus of control and Time 2 LMX-Affect (β = 0.06, *ns*) and Time 2 LMX-Professional respect (β = 0.06, *ns*).

## Discussion

Based on a two-wave design that controlled for the autoregressive effects of overall LMX or its dimensions, this study demonstrates that subordinates with an internal work locus of control develop social relationships of a higher quality with their supervisor over time. As such, the present study provides strong evidence that subordinates’ individual differences may act as major drivers of the development of LMX relationships. Moreover, this effect was found to be stronger when the work context offered the necessary conditions for such beneficial outcomes. Indeed, the relationship between internal work locus of control and LMX was stronger when individuals understood the expectations associated with their jobs (i.e., high role clarity). Such context allowed internals to be more confident in their ability to influence their environment, which enhanced their sense of agency. Using a dimensional approach to LMX, it was further found that only LMX’s loyalty dimension was fostered by the interaction between work locus of control and role clarity, suggesting that internals were particularly effective at building on conditions of clearly defined job responsibilities to obtain a behavioral engagement from supervisors to defend their goals in the organization. Overall, this study advances research by identifying how employees’ individual differences interact with features of the work context to build LMX relationships and which LMX dimensions are sensitive to this process. In the next sections, we discuss the implications of these findings for LMX research.

### Theoretical Implications

Our research contributes to the LMX literature primarily in three ways. First, we provide new insights into how LMX develops by exploring the role of individual differences (i.e., internal work locus of control) as antecedents of LMX, thereby delving into a neglected area of research on LMX (Martin et al., 2005; Schyns, 2015). As such, we are taking a step away from leader-centric approaches that have usually focused on leader characteristics and behaviors to explain the development of LMX (Dulebohn et al., 2012). Our findings are in line with previous research that has consistently reported a positive relationship between internal work locus of control and LMX using cross-sectional designs (e.g., Martin et al., 2005; Harris et al., 2007; Kauppila, 2014; Hao et al., 2019). However, cross-sectional designs are known to provide weak evidence regarding the directional nature of relationships among variables. Establishing temporal precedence among variables and eliminating plausible alternative explanations like reverse causality can be better achieved by using two-wave designs where dependent variables are measured twice, allowing their baseline level to be controlled for Antonakis et al. (2010). Using that approach, we were able to show that work locus of control acted as a driver of (change in) LMX relationships, thereby providing strong evidence that work locus of control temporally predicted how LMX relationships evolved over time. As such, our data provide support to the idea that dispositional characteristics such as work locus of control represent an important basis for the development of high-quality LMX relationships. In doing so, we heed the call of researchers who have invited researchers to use stronger designs (Dulebohn et al., 2012; Martin et al., 2016) in order to better understand how LMX relationships develop (van Breukelen et al., 2006), particularly as it comes to grasp the role of dispositional characteristics (Law et al., 2010).

Second, we highlight the importance of studying the contextual boundaries associated with the effects of work locus of control on LMX over time. This contextualized view is important to understand *when* these effects may take place. As such, our study shows that work locus of control influences LMX particularly well when role clarity is high. In contrast, when role expectations are unclear, the beneficial effects of an internal locus of control are hindered. Thus, the organizational context can help create the conditions that facilitate the development of high-quality LMX. Role clarity helps internals feel that they have control over, and the ability to act upon, their environment in order to establish constructive social exchange-based relationships with their supervisor. To the best of our knowledge, this study is the first to explore potential contextual boundaries associated with the relationship between work locus of control and LMX. In doing so, we extend prior research that has examined this relationship from a non-contextualized perspective and answer the call of prior researchers for considering moderators in the study of the effects of locus of control (Ng et al., 2006). Although the role of the work context has often been forgone in the LMX literature (Dulebohn et al., 2012), our research demonstrates that the context is an inherent element contributing to LMX development (van Breukelen et al., 2006). More broadly, the present study illustrates the value of adopting a combined person–situation approach to LMX relationships (Ozer, 2008).

Third, the present study offers a closer examination of LMX at the dimension level. Our results demonstrate that the main effect of work locus of control on change in LMX generalized to all dimensions of LMX (i.e., affect, loyalty, contribution, and professional respect), thereby revealing homologous relationships across construct and dimension levels of LMX (Wong et al., 2008). As such, employees with an internal work locus of control will be more prone to like, be loyal to, entertain professional respect perceptions of, and to be willing to help, their leader. This multidimensional approach uncovered that a specific “currency of exchange” (Law et al., 2010) may be more likely to emerge depending on the nature of the antecedents and contextual boundaries at play. Indeed, at the dimension level, only the loyalty dimension of LMX was impacted by the interaction between work locus of control and role clarity. Thus, when the work context allows internals to feel in control and to have a heightened sense of agency, they may be more inclined to demonstrate concrete behaviors of support toward their leader and have him or her engage in defending their interests and goals. This finding indicates that the adoption of a dimensional perspective to LMX can help identify which dimensions of the construct can be influenced by work locus of control as a function of the work context (i.e., clarity of role expectations).

Contrary to Hypothesis 3c, LMX’s contribution dimension was not responsive to the interaction between work locus of control and role clarity. Ergo, internals are more prone to invest energies in the dyad’s goals independently of the level of role clarity. This might be because, as research has shown, internals tend to have high task-related motivation (Henson and Beehr, 2018) and job performance (Ng et al., 2006; Wang et al., 2010), which may generalize across contexts. Thus, by taking a dimensional approach to LMX, we were able to disentangle the roles of different exchange components that underlie LMX to better understand the processes that create high-quality relationships with supervisors. Since most studies have adopted a unidimensional view of LMX (Dulebohn et al., 2017; Tse et al., 2018), it is plausible that researchers have missed specific dimension-level relationships that would have helped uncover the intricate antecedents and effects of LMX (Greguras and Ford, 2006; Wong et al., 2008; Tse et al., 2018). Multiple researchers (e.g., Zhou and Schriesheim, 2010; Schyns, 2015; Martin et al., 2016) have proposed that future research should differentiate among LMX dimensions and focus on specific exchange elements to gain a more accurate picture of how LMX relationships come about and how they affect organizational outcomes. As such, this study is an endeavor to take a step toward the multidimensional approach LMX researchers ought to take to further our understanding of leader–member relationships.

As it relates to the extent to which an individual is affected by external factors, the plasticity hypothesis (Brockner, 1988) brings an interesting perspective to our findings. This hypothesis stipulates that individuals with fewer internal resources (e.g., possessing an external work locus of control) react more strongly to cues from the external environment (e.g., role clarity) because the external context would provide needed resources (Fernet et al., 2010). As our argumentation suggests that internals are more responsive to the external environment (e.g., role clarity) because the external context allows them to gain control over events and eases their sense of agency, it is at odds with the plasticity hypothesis. Nonetheless, the plasticity hypothesis assumes that individuals differ in the importance they attribute to interpersonal relationships (Fernet et al., 2010) and on the extent to which they rely on external cues to derive their attitudes and behaviors (Pierce and Gardner, 2004). As such, internals, who may be less affected by their social environment (Pierce and Gardner, 2004), would rely more on their own dispositions to influence the context. This would explain why internals’ sense of agency plays an important role in the development of LMX relationships.

### Practical Implications

Given the obvious importance of an employee’s relationship with his or her supervisor, a richer understanding of the drivers and dynamics of this relationship can lead to better organizational practices. Maximizing efforts to promote the development of high-quality LMX relationships can lead to beneficial outcomes for individuals and organizations, including enhanced job satisfaction, organizational commitment, job performance, and reduced turnover (Dulebohn et al., 2012). Therefore, it may be beneficial to leaders to understand the nature of employees’ work locus of control (i.e., internal vs. external) and its effects. In doing so, either through feedback from survey results or verbal questioning, leaders may get this knowledge and build on it to make the best of employees’ personality. For example, they should make sure that employees with an internal work locus of control develop a clear understanding of their job responsibilities, which will help them gain the autonomy they desire to build a better relationship with the leader. Indeed, as shown in this study, ambiguity regarding role expectations hinders the potential benefits internals can get from the work context. Internals achieve better results in the workplace essentially because they invest time and energies to attain valued goals (Wang et al., 2010; Galvin et al., 2018). However, if they are not aware of what is expected of them and what means are available to achieve work goals, they may decide to flee such situations (Spector, 1982). Moreover, as work locus of control has been shown to have important motivational and attitudinal consequences, including enhanced LMX relationships in the current study, organizations would be well advised to include the assessment of this trait in recruitment and selection practices (Lam and Schaubroeck, 2000). For example, as internals possess a strong need for achievement (Spector, 1988; Galvin et al., 2018) and were shown in this study to develop stronger LMX relationships, organizations may benefit from hiring employees that exhibit an internal work locus of control. While doing so, organizations should be aware that providing these employees with clear expectations regarding their work role would increase the benefits of recruiting them. Similarly, the benefits of hiring internals would be increased in jobs requiring complex information processing (Spector, 1982) and in those where employees work closely with their supervisor when completing work duties.

On the other hand, one must not forsake employees with external work locus of control. As the present findings indicate, these people have a harder time developing high-quality LMX relationships. Presumably, their sense of agency is lower compared to internals, hence they are less likely to be confident that their behaviors can influence the relationship with the supervisor. Therefore, leaders may want to reinforce externals’ sense of control by empowering them and help them envision how their actions can alter their environment in a direction that results in achievable outcomes (e.g., task performance) (Lam and Schaubroeck, 2000). For instance, managers could provide more direct support to externals so as to help them maintain and develop constructive work relationships with supervisors, thereby instilling self-efficacy beliefs and the sense that they can obtain valued rewards and outcomes in the workplace (Lam and Schaubroeck, 2000). Managers should also be aware that they may have to invest more time and resources to instill a sense of control among externals. Similarly, leaders should ensure that externals are aware of the criteria and expectations they hold to help their relationship be constructive and grow over time. By clarifying how such contingencies ultimately lead to stronger performance, leaders would help externals be more confident in what actions can be done to foster LMX relationships. Moreover, as role clarity does not particularly help externals in developing LMX relationships, leaders should rely on their own communication efforts to build externals’ sense of agency (Lam and Schaubroeck, 2000; Ng et al., 2006). Such efforts should target externals’ understanding of the links between their own actions and desired outcomes.

### Limitations and Future Research Directions

Despite its strengths, this study is not without limitations. First, all measures were self-reported. Thus, results might be subject to common method variance (Podsakoff et al., 2012). However, interaction effects are known to be unaffected by a positive method bias (Siemsen et al., 2010), suggesting that the moderating effects of role clarity are robust. Still, we took steps to reduce method variance effects by collecting data at two separate times using a 6-month time lag and we controlled for baseline levels of our outcomes (i.e., overall LMX or LMX dimensions), which is known to considerably reduce endogeneity effects (Podsakoff et al., 2003; Ployhart and Vandenberg, 2010). Future extensions of this study could use similar longitudinal designs to include the consequences of LMX. It would indeed be interesting to examine the indirect effect of work locus of control on change in LMX outcomes, such as affective organizational commitment or even job performance, as well as the potential moderating effect of role clarity on these relationships. This would also allow exploring how LMX dimensions play out as distinct mediators between work locus of control and LMX outcomes.

Second, as a single source of data was used for LMX, only the employee’s perspective was considered, not the supervisor’s perception of LMX. As such assessments refer to dyadic relationships, it would be of interest to consider both partners’ views since supervisors and employees may evaluate the relationship using different aspects of LMX (Schyns and Wolfram, 2008; Zhou and Schriesheim, 2010). However, as we focused on the individual difference variable of work locus of control, we still believe that the employees’ point of view of the relationship was particularly important. The next step would be to use a supervisor-rated measure of LMX in order to grasp how supervisors’ perception of the relationship relates to, or is affected by, employees’ work locus of control. As such, further research could measure LMX from both perspectives to examine whether different dimensions of LMX are affected by the employee’s work locus of control across rating sources. Indeed, the potential divergence among supervisors’ and subordinates’ perceptions has been understudied in the LMX literature (Gooty and Yammarino, 2016). Since the level of agreement between rating sources may be lower vs. higher depending on the dimension of LMX that is considered (Sin et al., 2009), an interesting research avenue would also be to seek to identify which factors can explain diverging perceptions on LMX dimensions (Liden and Maslyn, 1998).

Third, by focusing on the individual characteristics of employees, this study has forgone how the individual traits of supervisors come into play. To have a more complete picture of the dyadic relationship, it would be worth exploring how the employees’ dispositions interact with supervisors’ dispositions since relatively little is known about the relative influence of leaders’ and followers’ traits in LMX development (Dulebohn et al., 2012). As some studies have begun to evaluate how the similarity and the compatibility among leaders’ and subordinates’ characteristics influence the quality of their relationships (e.g., Nahrgang and Seo, 2015), it might be insightful to examine how employees’ work locus of control interacts with supervisors’ own work locus of control to influence the development of LMX and how such interaction affects LMX outcomes (Galvin et al., 2018).

Fourth, while our sample included participants from multiple industries and various types of organizations, indicating that our results can be applicable to a large variety of jobs, there are some limitations to the generalizability of our results. For instance, our sample comprised 75% women. As we controlled for the baseline levels of the outcomes (e.g., LMX and its dimensions), thereby predicting change in the outcomes across time, potential confounding effects by gender (and other factors) are limited. However, it might be interesting to replicate our study to examine the generalizability of the results to the larger working population. Moreover, our data were collected in an individualistic country, which makes the generalizability of our results to collectivistic countries uncertain. Cultural values may indeed influence how individual characteristics are enacted and how employees’ relationships with their leader develop (House et al., 2004; Rockstuhl et al., 2012). For example, a culture with a performance orientation can represent a more thriving environment for internals because their need for achievement would be particularly valued. In contrast, a power distant culture may reduce opportunities for upward mobility, which may limit internals’ potential for getting ahead and make their sense of agency less effective. As power distance promotes respect for authority, and tends to be associated with a collectivistic culture, LMX relationships in such cultures may be more affected by role-based loyalty and obligations (Rockstuhl et al., 2012). Thus, individual dispositions (i.e., work locus of control) may be less relevant to the development of high LMX relationships in collectivistic cultures. Further inquiry is warranted to understand how LMX relationships develop in different cultures.

## Data Availability Statement

The datasets generated for this study are available on request to the corresponding author.

## Ethics Statement

The studies involving human participants were reviewed and approved by the Research Ethics Board (REB) of HEC Montreal. The patients/participants provided their written informed consent to participate in this study.

## Author Contributions

CV designed the study. VR collected the data. Both authors developed the theoretical framework for the study, wrote the manuscript altogether, collaborated in developing the manuscript, read and approved the submitted version, and performed the data analyses.

## Conflict of Interest

The authors declare that the research was conducted in the absence of any commercial or financial relationships that could be construed as a potential conflict of interest.

## References

[B1] Aiken L. S. West S. G. (1991). *Multiple Regression: Testing and Interpreting Interactions.* Newbury Park, CA: Sage Publications.

[B2] Allen D. G. Weeks K. P. Moffitt K. R. (2005). Turnover intentions and voluntary turnover: the moderating roles of self-monitoring, locus of control, proactive personality, and risk aversion. *J. Appl. Psychol.* 90 980–990. 10.1037/0021-9010.90.5.980 16162070

[B3] Anand S. Hu J. Liden R. C. Vidyarthi P. R. (2011). “Leader-member exchange: recent research findings and prospects for the future,” in *The SAGE Handbook of Leadership*, eds Bryman A. Collinson D. L. Grint K. Jackson B. Uhl-Bien M. (London: Sage Publications), 311–325.

[B4] Anand S. Vidyarthi P. Rolnicki S. (2018). Leader-member exchange and organizational citizenship behaviors: contextual effects of leader power distance and group task interdependence. *Leadersh. Q.* 29 489–500. 10.1016/j.leaqua.2017.11.002

[B5] Anand S. Vidyarthi P. R. Liden R. C. Rousseau D. M. (2010). Good citizens in poor-quality relationships: idiosyncratic deals as a substitute for relationship quality. *Acad. Manage. J.* 53 970–988. 10.5465/amj.2010.54533176

[B6] Antonakis J. Bendahan S. Jacquart P. Lalive R. (2010). On making causal claims: a review and recommendations. *Leadersh. Q.* 21 1086–1120. 10.1016/j.leaqua.2010.10.010

[B7] Bentler P. M. Bonett D. G. (1980). Significance tests and goodness of fit in the analysis of covariance structures. *Psychol. Bull.* 88 588–606. 10.1037/0033-2909.88.3.588

[B8] Blau P. M. (1964). *Exchange and Power in Social Life.* New York, NY: John Wiley & Sons.

[B9] Brockner J. (1988). *Self-Esteem at Work: Research, Theory, and Practice.* Lexington, MA: Lexington Books.

[B10] Dansereau F. Jr. Graen G. Haga W. J. (1975). A vertical dyad linkage approach to leadership within formal organizations: a longitudinal investigation of the role making process. *Organ. Behav. Hum. Perform.* 13 46–78. 10.1016/0030-5073(75)90005-7

[B11] Dawson J. F. (2014). Moderation in management research: what, why, when, and how. *J. Bus. Psychol.* 29 1–19. 10.1007/s10869-013-9308-7

[B12] Dienesch R. M. Liden R. C. (1986). Leader-member exchange model of leadership: a critique and further development. *Acad. Manage. Rev.* 11 618–634. 10.5465/amr.1986.4306242

[B13] Dulebohn J. H. Bommer W. H. Liden R. C. Brouer R. L. Ferris G. R. (2012). A meta-analysis of antecedents and consequences of leader-member exchange: integrating the past with an eye toward the future. *J. Manage.* 38 1715–1759. 10.1177/0149206311415280

[B14] Dulebohn J. H. Wu D. Liao C. (2017). Does liking explain variance above and beyond LMX? A meta-analysis. *Hum. Resour. Manage. Rev.* 27 149–166. 10.1016/j.hrmr.2016.09.008

[B15] Erdogan B. Enders J. (2007). Support from the top: supervisors’ perceived organizational support as a moderator of leader-member exchange to satisfaction and performance relationships. *J. Appl. Psychol.* 92 321–330. 10.1037/0021-9010.92.2.321 17371081

[B16] Fernet C. Gagné M. Austin S. (2010). When does quality of relationships with coworkers predict burnout over time? The moderating role of work motivation. *J. Organ. Behav.* 31 1163–1180. 10.1002/job.673

[B17] Galvin B. M. Randel A. E. Collins B. J. Johnson R. E. (2018). Changing the focus of locus (of control): a targeted review of the locus of control literature and agenda for future research. *J. Organ. Behav.* 39 820–833. 10.1002/job.2275

[B18] Geiser C. (2012). *Data Analysis with Mplus.* New York, NY: Guilford press 10.1007/978-3-531-93192-0

[B19] Gerstner C. R. Day D. V. (1997). Meta-analytic review of leader–member exchange theory: correlates and construct issues. *J. Appl. Psychol.* 82 827–844. 10.1037/0021-9010.82.6.827

[B20] Goodman J. S. Blum T. C. (1996). Assessing the non-random sampling effects of subject attrition in longitudinal research. *J. Manage.* 22 627–652. 10.1177/014920639602200405

[B21] Gooty J. Yammarino F. J. (2016). The leader–member exchange relationship: a multisource, cross-level investigation. *J. Manage.* 42 915–935. 10.1177/0149206313503009

[B22] Graen G. Cashman J. F. (1975). “A role-making model of leadership in formal organizations: A developmental approach,” in *Leadership frontiers*, eds Hung J. G. Larson L. L. (Kent, OH: Kent State University Press), 143–165.

[B23] Graen G. Scandura T. A. (1987). “Toward a psychology of dyadic organizing,” in *Research in Organizational Behavior*, Vol. 9 eds Staw B. Cummings L. L. (Greenwich, CT: JAI Press), 175–208.

[B24] Graen G. Uhl-Bien M. (1995). Relationship-based approach to leadership: development of leader-member exchange (LMX) theory of leadership over 25 years: applying a multi-level multi-domain perspective. *Leadersh. Q.* 6 219–247. 10.1016/1048-9843(95)90036-5

[B25] Gregersen S. Vincent-Höper S. Nienhaus A. (2016). Job-related resources, leader–member exchange and well-being: a longitudinal study. *Work Stress* 30 356–373. 10.1080/02678373.2016.1249440

[B26] Greguras G. J. Ford J. M. (2006). An examination of the multidimensionality of supervisor and subordinate perceptions of leader-member exchange. *J. Occup. Organ. Psychol.* 79 433–465. 10.1348/096317905X53859 30467716

[B27] Hao Q. Shi Y. Yang W. (2019). How leader-member exchange affects knowledge sharing behavior: understanding the effects of commitment and employee characteristics. *Front. Psychol.* 10:2768. 10.3389/fpsyg.2019.02768 31920820 PMC6914851

[B28] Harris K. J. Harris R. Eplion D. (2007). Personality, leader-member exchanges, and work outcomes. *J. Behav. Appl. Manage.* 8 92–107. 10.21818/001c.16715

[B29] Harris K. J. Kacmar K. M. Carlson D. S. (2006). An examination of temporal variables and relationship quality on promotability ratings. *Group Organ. Manage.* 31 677–699. 10.1177/1059601106286889

[B30] Henson J. A. Beehr T. (2018). Subordinates’ core self-evaluations and performance predict leader-rated LMX. *Leadersh. Organ. Dev. J.* 39 150–168. 10.1108/LODJ-06-2016-0162

[B31] House R. J. Hanges P. J. Javidan M. Dorfman P. W. Gupta V. (2004). *Culture, Leadership, and Organizations: The GLOBE Study of 62 Societies.* Thousand Oaks, CA: Sage Publications.

[B32] Johnson R. E. Rosen C. C. Chang C.-H. Lin S.-H. (2015). Getting to the core of locus of control: Is it a core evaluation of the self or the environment? *J. Appl. Psychol.* 100 1568–1578. 10.1037/apl0000011 25664470

[B33] Kahn R. L. Wolfe D. M. Quinn R. P. Snoek D. Jr. Rosenthal R. A. (1964). *Organizational Stress: Studies in Role Conflict and Ambiguity.* New York, NY: Wiley.

[B34] Katz D. Kahn R. (1978). *The Social Psychology of Organizations*, 2nd Edn New York, NY: Wiley.

[B35] Kauppila O. P. (2014). So, what am I supposed to do? A multilevel examination of role clarity. *J. Manage. Stud.* 51 737–763. 10.1111/joms.12042

[B36] Lam S. S. Schaubroeck J. (2000). The role of locus of control in reactions to being promoted and to being passed over: a quasi experiment. *Acad. Manage. J.* 43 66–78. 10.5465/1556386

[B37] Law K. S. Wang H. Hui C. (2010). Currencies of exchange and global LMX: how they affect employee task performance and extra-role performance. *Asia Pac. J. Manage.* 27 625–646. 10.1007/s10490-009-9141-8

[B38] Lee J. (2005). Effects of leadership and leader-member exchange on commitment. *Leadersh. Organ. Dev. J.* 26 655–672. 10.1108/01437730510633728

[B39] Lefcourt H. M. (1976). Locus of control and the response to aversive events. *Can. Psychol. Rev.* 17 202–209. 10.1037/h0081839

[B40] Liden R. C. Graen G. (1980). Generalizability of the vertical dyad linkage model of leadership. *Acad. Manage. J.* 23 451–465. 10.5465/255511

[B41] Liden R. C. Maslyn J. M. (1998). Multidimensionality of leader-member exchange: an empirical assessment through scale development. *J. Manage.* 24 43–72. 10.1016/S0149-2063(99)80053-1

[B42] Liden R. C. Sparrowe R. T. Wayne S. J. (1997). “Leader-member exchange theory: the past and potential for the future,” in *Research in Personal and Human Resources Management*, Vol. 15 ed. Ferris G. R. (Greenwich, CT: JAI Press Inc), 47–119.

[B43] Liden R. C. Wayne S. J. Stilwell D. (1993). A longitudinal study on the early development of leader-member exchanges. *J. Appl. Psychol.* 78 662–674. 10.1037/0021-9010.78.4.662

[B44] Lievens F. De Corte W. Schollaert E. (2008). A closer look at the frame-of-reference effect in personality scale scores and validity. *J. Appl. Psychol.* 93 268–279. 10.1037/0021-9010.93.2.268 18361631

[B45] Little T. D. Rhemtulla M. Gibson K. Schoemann A. M. (2013). Why the items versus parcels controversy needn’t be one. *Psychol. Methods* 18 285–300. 10.1037/a0033266 23834418 PMC3909043

[B46] Lord R. G. Gatti P. Chui S. L. (2016). Social-cognitive, relational, and identity-based approaches to leadership. *Organ. Behav. Hum. Decis. Process.* 136 119–134. 10.1016/j.obhdp.2016.03.001

[B47] Martin R. Guillaume Y. Thomas G. Lee A. Epitropaki O. (2016). Leader-member exchange (LMX) and performance: a meta-analytic review. *Pers. Psychol.* 69 67–121. 10.1111/peps.12100

[B48] Martin R. Thomas G. Charles K. Epitropaki O. McNamara R. (2005). The role of leader-member exchanges in mediating the relationship between locus of control and work reactions. *J. Occup. Organ. Psychol.* 78 141–147. 10.1348/096317904X23763 30467716

[B49] Maslyn J. M. Uhl-Bien M. (2001). Leader–member exchange and its dimensions: effects of self-effort and other’s effort on relationship quality. *J. Appl. Psychol.* 86 697–708. 10.1037/0021-9010.86.4.697 11519653

[B50] Muthén L. K. Muthén B. O. (2010). *Mplus User’s Guide, version 6.1.* Los Angeles, CA: Muthén & Muthén.

[B51] Nahrgang J. D. Seo J. J. (2015). “How and why high leader–member exchange (LMX) relationships develop: examining the antecedents of LMX,” in *The Oxford Handbook of Leader-Member Exchange*, eds Bauer T. N. Erdogan B. (Oxford: Oxford University Press), 87–118. 10.1093/oxfordhb/9780199326174.013.0003

[B52] Newman A. Allen B. Miao Q. (2015). I can see clearly now: the moderating effects of role clarity on subordinate responses to ethical leadership. *Pers. Rev.* 44 611–628. 10.1108/PR-11-2013-0200

[B53] Ng T. W. Eby L. T. Sorensen K. L. Feldman D. C. (2005). Predictors of objective and subjective career success: a meta-analysis. *Pers. Psychol.* 58 367–408. 10.1111/j.1744-6570.2005.00515.x

[B54] Ng T. W. Sorensen K. L. Eby L. T. (2006). Locus of control at work: a meta-analysis. *J. Organ. Behav.* 27 1057–1087. 10.1002/job.416

[B55] Ozer M. (2008). Personal and task-related moderators of leader-member exchange among software developers. *J. Appl. Psychol.* 93 1174–1182. 10.1037/0021-9010.93.5.1174 18808235

[B56] Panaccio A. Vandenberghe C. (2011). The relationships of role clarity and organization-based self-esteem to commitment to supervisors and organizations and turnover intentions. *J. Appl. Soc. Psychol.* 41 1455–1485. 10.1111/j.1559-1816.2011.00764.x

[B57] Pierce J. L. Gardner D. G. (2004). Self-esteem within the work and organizational context: a review of the organization-based self-esteem literature. *J. Manage.* 30 591–622. 10.1016/j.jm.2003.10.001

[B58] Ployhart R. E. Vandenberg R. J. (2010). Longitudinal research: the theory, design, and analysis of change. *J. Manage.* 36 94–120. 10.1177/0149206309352110

[B59] Podsakoff P. M. MacKenzie S. B. Lee J.-Y. Podsakoff N. P. (2003). Common method biases in behavioral research: a critical review of the literature and recommended remedies. *J. Appl. Psychol.* 88 879–903. 10.1037/0021-9010.88.5.879 14516251

[B60] Podsakoff P. M. MacKenzie S. B. Podsakoff N. P. (2012). Sources of method bias in social science research and recommendations on how to control it. *Annu. Rev. Psychol.* 63 539–569. 10.1146/annurev-psych-120710-100452 21838546

[B61] Preacher K. J. Curran P. J. Bauer D. J. (2006). Computational tools for probing interaction effects in multiple linear regression, multilevel modeling, and latent curve analysis. *J. Educ. Behav. Stat.* 31 437–448. 10.3102/10769986031004437

[B62] Rizzo J. R. House R. J. Lirtzman S. I. (1970). Role conflict and ambiguity in complex organizations. *Adm. Sci. Q.* 15 150–163. 10.2307/2391486

[B63] Rockstuhl T. Dulebohn J. H. Ang S. Shore L. M. (2012). Leader–member exchange (LMX) and culture: a meta-analysis of correlates of LMX across 23 countries. *J. Appl. Psychol.* 97 1097–1130. 10.1037/a0029978 22985117

[B64] Rotter J. B. (1954). *Social Learning and Clinical Psychology.* Englewood Cliffs, NJ: Prentice-Hall 10.1037/10788-000

[B65] Rotter J. B. (1966). Generalized expectancies for internal versus external control of reinforcement. *Psychol. Monogr.* 80 1–28. 10.1037/h0092976 5340840

[B66] Scandura T. A. Schriesheim C. A. (1994). Leader-member exchange and supervisor career mentoring as complementary constructs in leadership research. *Acad. Manage. J.* 37 1588–1602. 10.2307/256800

[B67] Schaffer B. S. Riordan C. M. (2003). A review of cross-cultural methodologies for organizational research: a best-practices approach. *Organ. Res. Methods* 6 169–215. 10.1177/1094428103251542

[B68] Schriesheim C. A. Castro S. L. Cogliser C. C. (1999). Leader-member exchange (LMX) research: a comprehensive review of theory, measurement, and data-analytic practices. *Leadersh. Q.* 10 63–113. 10.1016/S1048-9843(99)80009-5

[B69] Schyns B. (2015). “Leader and follower personality and LMX,” in *The Oxford Handbook of Leader-Member Exchange*, eds Bauer T. N. Erdogan B. (Oxford: Oxford University Press). 10.1093/oxfordhb/9780199326174.013.0016

[B70] Schyns B. Wolfram H.-J. (2008). The relationship between leader-member exchange and outcomes as rated by leaders and followers. *Leadersh. Organ. Dev. J.* 29 631–646. 10.1108/01437730810906362

[B71] Siemsen E. Roth A. Oliveira P. (2010). Common method bias in regression models with linear, quadratic, and interaction effects. *Organ. Res. Methods* 13 456–476. 10.1177/1094428109351241

[B72] Sin H. P. Nahrgang J. D. Morgeson F. P. (2009). Understanding why they don’t see eye to eye: an examination of leader–member exchange (LMX) agreement. *J. Appl. Psychol.* 94 1048–1057. 10.1037/a0014827 19594243

[B73] Sparrowe R. T. Liden R. C. (2005). Two routes to influence: integrating leader-member exchange and social network perspectives. *Adm. Sci. Q.* 50 505–535. 10.2189/asqu.50.4.505 21821037

[B74] Spector P. E. (1982). Behavior in organizations as a function of employees’ locus of control. *Psychol. Bull.* 91 482–497. 10.1037/0033-2909.91.3.482

[B75] Spector P. E. (1988). Development of the work locus of control scale. *J. Occup. Psychol.* 61 335–340. 10.1111/j.2044-8325.1988.tb00470.x

[B76] Tse H. H. M. Troth A. C. Ashkanasy N. M. Collins A. L. (2018). Affect and leader-member exchange in the new millennium: a state-of-art review and guiding framework. *Leadersh. Q.* 29 135–149. 10.1016/j.leaqua.2017.10.002

[B77] Tubre T. C. Collins J. M. (2000). Jackson and Schuler (1985) revisited: a meta-analysis of the relationships between role ambiguity, role conflict, and job performance. *J. Manage.* 26 155–169. 10.1177/014920630002600104

[B78] van Breukelen W. Schyns B. Le Blanc P. (2006). Leader-member exchange theory and research: accomplishments and future challenges. *Leadership* 3 295–316. 10.1177/1742715006066023

[B79] Vidyarthi P. R. Liden R. C. Anand S. Erdogan B. Ghosh S. (2010). Where do I stand? Examining the effects of leader–member exchange social comparison on employee work behaviors. *J. Appl. Psychol.* 95 849–861. 10.1037/a0020033 20718513

[B80] Wang D. Gan C. Wu C. (2016). LMX and employee voice: a moderated mediation model of psychological empowerment and role clarity. *Pers. Rev.* 45 605–615. 10.1108/PR-11-2014-0255

[B81] Wang Q. Bowling N. A. Eschleman K. J. (2010). A meta-analytic examination of work and general locus of control. *J. Appl. Psychol.* 95 761–768. 10.1037/a0017707 20604595

[B82] Wayne S. J. Liden R. C. Kraimer M. L. Graf I. K. (1999). The role of human capital, motivation and supervisor sponsorship in predicting career success. *J. Organ. Behav.* 20 577–595. 10.1002/(SICI)1099-1379(199909)20:5<577::AID-JOB958>3.0.CO;2-0

[B83] Wayne S. J. Shore L. M. Liden R. C. (1997). Perceived organizational support and leader-member exchange: a social exchange perspective. *Acad. Manage. J.* 40 82–111. 10.5465/257021

[B84] Webster J. R. Beehr T. A. (2013). Antecedents and outcomes of employee perceptions of intra-organizational mobility channels. *J. Organ. Behav.* 34 919–941. 10.1002/job.1823

[B85] Wong C. S. Law K. S. Huang G. H. (2008). On the importance of conducting construct-level analysis for multidimensional constructs in theory development and testing. *J. Manage.* 34 744–764. 10.1177/0149206307312506

[B86] Yukl G. A. Latham G. P. (1978). Interrelationships among employee participation, individual differences, goal difficulty, goal acceptance, goal instrumentality, and performance. *Pers. Psychol.* 31 305–323. 10.1111/j.1744-6570.1978.tb00449.x

[B87] Zhou X. T. Schriesheim C. A. (2010). Quantitative and qualitative examination of propositions concerning supervisor-subordinate convergence in descriptions of leader-member exchange (LMX) quality. *Leadersh. Q.* 21 826–843. 10.1016/j.leaqua.2010.07.010

